# A spatial epidemiological analysis of nontuberculous mycobacterial infections in Queensland, Australia

**DOI:** 10.1186/1471-2334-14-279

**Published:** 2014-05-21

**Authors:** Michael P Chou, Archie CA Clements, Rachel M Thomson

**Affiliations:** 1 University of Queensland, Infectious Disease Epidemiology Unit, School of Population Health, Brisbane, Australia; 2 Gallipoli Medical Research Centre, Greenslopes Private Hospital, Brisbane, Australia; 3 CHRISP-TB, Queensland Health, Brisbane, Australia; 4 School of Population Health, University of Queensland, Level 2, Public Health Building, Herston Road, Herston, QLD 4006, Australia

**Keywords:** Nontuberculous mycobacteria (NTM), *Mycobacterium intracellulare*, *Mycobacterium kansasii*, *Mycobacterium abscessus*, Spatial epidemiology

## Abstract

**Background:**

The epidemiology of infections with nontuberculous mycobacteria (NTM) has been changing and the incidence has been increasing in some settings. The main route of transmission to humans is considered to be from the environment. We aimed to describe spatial clusters of cases of NTM infections and to identify associated climatic, environmental and socio-economic variables.

**Methods:**

NTM data were obtained from the Queensland Mycobacterial Reference Laboratory for the period 2001–2011. A Bayesian spatial conditional autoregressive model was constructed at the postcode level, with covariates including soil variables, maximum, mean and minimum rainfall and temperature, income (proportion of population earning < $32,000 and < $52,000) and land use category.

**Results:**

Significant clusters of NTM infection were identified in the central Queensland region overlying the Surat sub-division of the Great Artesian Basin, as well as in the lower North Queensland Local Government Area known as the Whitsunday region. Our models estimated an expected increase of 21% per percentage increase of population earning < $52,000 (95% CI 9–34%) and an expected decrease of 13% for every metre increase of average topsoil depth for risk of *Mycobacterium intracellulare* infection (95% CI -3 – -22%). There was an estimated increase of 79% per mg/m^3^ increase of soil bulk density (95% CI 26–156%) and 19% decrease for every percentage increase in population earning < $32,000 for risk of *M. kansasii* infection (95% CI -3 – -49%).

**Conclusions:**

There were distinct spatial clusters of *M. kansasii, M. intracellulare* and *M. abscessus* infections in Queensland, and a number of socio-ecological, economic and environmental factors were found to be associated with NTM infection risk.

## Background

Nontuberculous mycobacteria (NTM) are ubiquitous in environments around the world. NTM have been isolated from soil, water and air as well as in more engineered contexts like water pipes, showerheads, bio-films and transgenic plants [1,2]. Interest in NTM has been gaining steadily over the past 20 years as associations between NTM and HIV/AIDS, and NTM and structural lung diseases, have become better understood [3].

NTM incidence has been shown to be high in particular geographic regions and ecologies [4]. In Australia and the United States, NTM isolates are more common than *Mycobacterium tuberculosis* (TB) isolates. Incidence rates for NTM are estimated to be between 1.0–1.8 per 100,000 persons per year in developed countries [3]. HIV infection predisposes a host to NTM infection and the HIV epidemic has been an important contributor to increased incidence of NTM infections in developed countries [5].

There remain areas of uncertainty in the characterisation of NTM epidemiology and pathogenicity because NTM contamination and colonisation from the environment is common and not necessarily harmful [6]. Furthermore, clinical reporting is often not uniformly standardised. Therefore, complete, high-quality epidemiological data on NTM infections are not always available. Fortunately, the Australian state of Queensland has had a mandatory reporting scheme for NTM infections since the introduction of a nationwide TB control campaign. All isolates of NTM throughout the state are recorded in a database, providing unique opportunities for epidemiological research.

With increasing availability of data online and accessibility of geographic information system (GIS) software and other mapping tools, spatial analytical techniques in epidemiology have become well established. Spatial methods have been employed in disease mapping, health surveillance and the development of spatial decision support systems [7]. Spatial analysis facilitates the identification of regions with high disease incidence and provides justification for more targeted investigations or interventions. A previous study examined spatial clusters of NTM infections in the United States (US) and identified higher income and education levels, higher evapotranspiration of surface water and higher copper and sodium soil levels as significant predictors of NTM infection risk [8]. This study from the U.S. supports the need to further investigate environmental predictors and spatial patterns of NTM in different settings.

The epidemiology of NTM infection is changing and refining the understanding of disease predictors will better inform clinical and public health efforts to control the disease. This paper aims to identify spatial patterns of NTM infections in Queensland between 2001 and 2011, and the association between NTM infection risk and a range of climate, environmental, demographic and socio-economic variables.

## Methods

### Study area

Queensland is located in the northeast of Australia between the latitudes of 10°S and 28°S and longitudes of 138°E and 153°E. The population of Queensland is 4.6 million people, and the state is administratively divided into 74 local government areas and 423 postcodes. Key industries include tourism, agriculture and mining. Climactically, northern Queensland is tropical and southern Queensland is sub-tropical. Most of the state experiences two distinct weather seasons: a warm summer with higher rainfall and a mild winter with lower rainfall.

### Ethical clearance

The project was approved by the Research Ethics committee of the University of Queensland (Project number 2012000471) in accordance with the provisions contained in the National Statement on Ethical Conduct in Human Research.

### Data sources

#### Nontuberculous mycobacterial infection data

All public hospital patient specimens for which mycobacterial culture is requested are forwarded to the Queensland Mycobacterial Reference Laboratory (QMRL). Requests in the private sector are processed by two main laboratories (providing >90% of the private mycobacterial pathology service in the state) and a few smaller laboratories. The two main private laboratories identify mycobacteria in specimens and, if detected, report them as “Atypical mycobacteria, not further specified”. Subsequent to culture, not all isolates are forwarded to the QMRL for species identification. However, the private laboratories do send isolates when (i) the specimen is smear positive (to exclude TB), (ii) the specimen is a bronchial washing or was obtained from a site other than sputum, (iii) there is more than one specimen positive for that patient, or (iv) at the request of the treating clinician. The majority of nonpulmonary isolates grown by private laboratories are sent to the QMRL for speciation. Hence, all speciated and the majority of unspeciated NTM are included in the database of the QLD TB control centre.

In terms of the specific laboratory methodology, human samples were digested and decontaminated using 4% NaOH, neutralised with phosphoric acid and centrifuged at 3000 g to concentrate the acid-fast bacilli (AFB). Smears were prepared from the sediment and stained by the Ziehl-Niehlsen (ZN) method. One Lowenstein-Jensen slope (pyruvate) and 7 ml Mycobacterial Growth Indicator Tube (MGIT) were inoculated and incubated at 35°C until growth was detected. ZN staining of colonies confirmed AFB. Multiplex PCR was performed to discriminate between *M. tuberculosis*, *M. avium*, *M. intracellulare*, *M. abscessus* and other *Mycobacterium* spp [9]. Isolates identified as other *Mycobacterium* spp were further speciated using Hain Life Sciences GenoType Mycobacterium AS (additional species) kit (2004-7 only) and/or 16S rRNA sequencing in conjunction with phenotypic characteristics.

For this study, all notified isolates from 2001–2011 were retrieved for analysis. In the case of multiple cultures per patient, only one isolate per calendar year was included, unless multiple species were identified. In this way, a reasonable balance may be achieved between capturing the environmental burden of strain types with the over-representation of patients with relapsed or persistent disease. For those with the same species - mainly *M. intracellulare -* it has been shown that most cases of subsequent isolation of MAC in individual patients represent reinfection with different strains, as opposed to relapse. [10] Age at time of positive isolate, gender and residential address were retrieved from de-identified records in the QTBCC database.

#### Demographic and environmental data

Income data were obtained from the 2010–11 Australian Bureau of Statistics [11]. The income variables percentage < $32 000 and percentage < $52 000 (of annual income) were based on the classifications of the Australian Bureau of Statistics. Percentage < $32 000 reflected relative disadvantage and < $52 000 is related to the average annual income of $51 923 for Australians in 2010–11. Indigenous and labour data were obtained from the Office of Economic and Statistical Research [12]. Thirty arc-second resolution raster data for maximum, minimum and mean temperature and rainfall were obtained from the WorldClim database [13]. Data on soil characteristics (permeability, depth, drainage, pH, nitrogen, phosphorus, bulk density, and composition), with a 1 km resolution, were obtained from the Commonwealth Scientific and Industrial Research Organisation (CSIRO) Australian Soil Resource Information System [14]. Layer A (surface layer, or topsoil) variables were used because this layer interfaces directly with human contact and activity. Soil composition classification data were refined for categorical analysis into their predominant base soil types - sand, clay, peat and loam. Landuse data were obtained from the national scale land use version 4 data (2005–2006) of the Australian Collaborative Landuse and Management Program (ACLUMP) [15]. Landuse was classified using six broad categories: conservation and natural environments; grazing vegetation and forestry production; dryland agriculture and plantations; irrigated agriculture and plantations; intensive uses/industry; and water.

Each of the demographic and environmental variables was summarized by postcode. For the environmental variables available in raster format, this involved extracting the mean value of the pixels that fell within the polygon that defined the boundary of the postcode. Extractions were done using the geographical information system (GIS) software ArcGIS (version 10.1).

### Statistical analysis

For selected species of NTM (*M. abscessus, M. avium, M. chelonae, M. fortuitum, M. gordonae, M. intracellulare, M. kansasii*), age and sex-adjusted standardised morbidity ratios (SMR) were calculated by postcode. Pairwise Spearman’s correlation coefficients were calculated for each pair of continuous predictor variables, and if the correlation coefficient was >0.9, the variable with the highest p-value in a bivariate Poisson regression model was excluded from further analysis.

For each of the selected species of NTM, the number of cases recorded by postcode during 2001–2011 was denoted as the dependent variable in an initial Poisson regression analysis, conducted using the statistical software package Stata version 11.2 (Statacorp, College Station, Texas). The expected number of cases for each postcode, adjusted for the age (categorised as <65 years and ≥65 years) and sex distribution of each postcode (using 2011 census data from the Australian Bureau of Statistics), was calculated, and entered into the models as an offset. Saturated models were created using all of the demographic and environmental variables as covariates (excluding the collinear variables) and an iterative, backwards stepwise method was used to reduce the set of covariates to those that were significantly associated with the dependent variable (*p* < 0.05).

Following covariate selection, Poisson and zero-inflated Poisson models were compared using the Akiake Information Criterion and Vuong Statistic (notably, at least 30% of the postcodes for each NTM species had zero incidence). Based on the model results, the standard form of Poisson regression was adopted for subsequent analyses.

For each NTM species, three mixed-effects Poisson regression models were created in a Bayesian framework using the statistical software WinBUGS (version 1.43). The first model (Model I) included the selected covariates with an unstructured postcode-level random effect. The second model (Model II) included the selected covariates with a spatially structured random effect. The third model (Model III), a convolution model, included the covariates and both the unstructured and spatially structured random effects.

Because a different set of covariates was used for each NTM species, the following shows a generalised form of Model III with *n* predictor variables, under the assumption that the observed counts of each NTM species (*Y*_*i*_) for the *i* th postcode followed a Poisson distribution, with mean *μ*:

$$\begin{array}{l} Y_{i}\approx \mathrm{Poisson}\left(\mu_{i}\right)\log\left(\mu_{i}\right)\;=\;\log(E_{i})+\theta_{i}\theta_{i}\\ \;=\alpha +\beta_{1}\left(\mathrm{Variable}\_1_{i}\right)\\ \;+B_{2}(\mathrm{Variable}\_2_{i})+\ldots \\ \;+B_{n}(\mathrm{Variable}\_n_{i})+w_{i}+s_{i} \end{array}$$

where *E*_*i*_ is the age and sex-adjusted expected number of cases in postcode *i,* α represents the intercept, β_1_…β_n_, are the coefficients for the covariates, *w*_*i*_ are the unstructured random effects, with a mean of zero and variance $\sigma_{w}^{2}$ and *s*_*i*_ are the spatially structured random effects, with a mean of zero and variance $\sigma_{s}^{2}$. Models I and II simply excluded *s*_*i*_ and *w*_*i*_ respectively.

Priors for the spatially structured random effects were derived using an intrinsic conditional autoregressive (CAR) modelling structure with an adjacency weights matrix of the postcodes. The adjacency matrix was constructed using the maps2WinBUGS plugin (version 2.2) of Quantum GIS (QGIS Wroclaw, version 1.73); postcode pairs that were neighbours were given a weight of 1 and postcode pairs that were not neighbours were given a weight of 0. Priors for the coefficients were assumed to have normal distributions (with mean = 0 and 1/variance = 1 × 10^-4^). A flat prior (i.e. an unbounded uniform distribution) was adopted for the intercept. The variance parameters for the random effects were assumed to have inverse gamma distributions, with shape and scale parameter values of 0.5 and 0.005 respectively.

A Bayesian imputation approach was employed to account for missing data among the covariates. There were at most two missing values from each set of 423 values (per variable). All variables with missing values were related to soil or landuse. Missing values were included as random variables. For the categorical covariates (included in the model as binary dummy variables), the random variables were assumed to have a Bernoulli distribution with a uniform prior ranging from 0 to 1, while missing continuous data were assumed to have a normal distribution with non-informative priors for both the mean and precision.

Using the Bayesian approach, posterior distributions for the random variables were estimated using Markov chain Monte Carlo simulation with Gibbs sampling employed by WinBUGS. Models were run with two chains. The initial 3000 iterations of the chains were discarded as burn in. Posterior kernel densities and history plots were visualised to assess for convergence and mixing. The models were subsequently thinned as appropriate based on inspection of autocorrelation plots. Depending on the factor for thinning, the chains were run until 20,000 samples were obtained for parameter estimation once convergence was observed. The Deviance Information Criterion (DIC) for each model was also monitored for model comparison and selection.

## Results

Over the 10-year period, there were a total of 6,599 NTM isolate notifications. Table 1 shows descriptive statistics for the most frequently isolated NTM species.

**Table 1:** Descriptive statistics for NTM isolates from 2001-2011

**Species**	**Count**			**Proportion of all isolates**	**Mean ± SD (per postcode)**	**Range**
	**Total**	**Pulmonary**	**Extra- pulmonary**			
*M. intracellulare*	2,306	2,184	122	34.9%	5.50 ± 7.76	0–63
*M. avium*	674	599	75	10.2%	1.50 ± 2.72	0–16
*M. fortuitum*	493	195	298	7.5%	1.10 ± 1.92	0–13
*M. abscessus*	489	334	155	7.4%	1.16 ± 2.01	0–17
*M. kansasii*	188	174	14	2.8%	0.45 ± 1.17	0–15
*M. chelonae*	183	64	119	2.8%	0.43 ± 0.95	0–9
*M. gordonae*	162	146	16	2.5%	0.39 ± 0.81	0–6
*Other*	1,357	909	448	20.6%	2.40 ± 3.61	0-34
*Unspeciated*	747	703	44	11.3%	2.39 ± 3.59	0-31
*Total*	6,599	5308	1291	100.0%	2.55 ± 4.96	0-63

**Figure 1:**
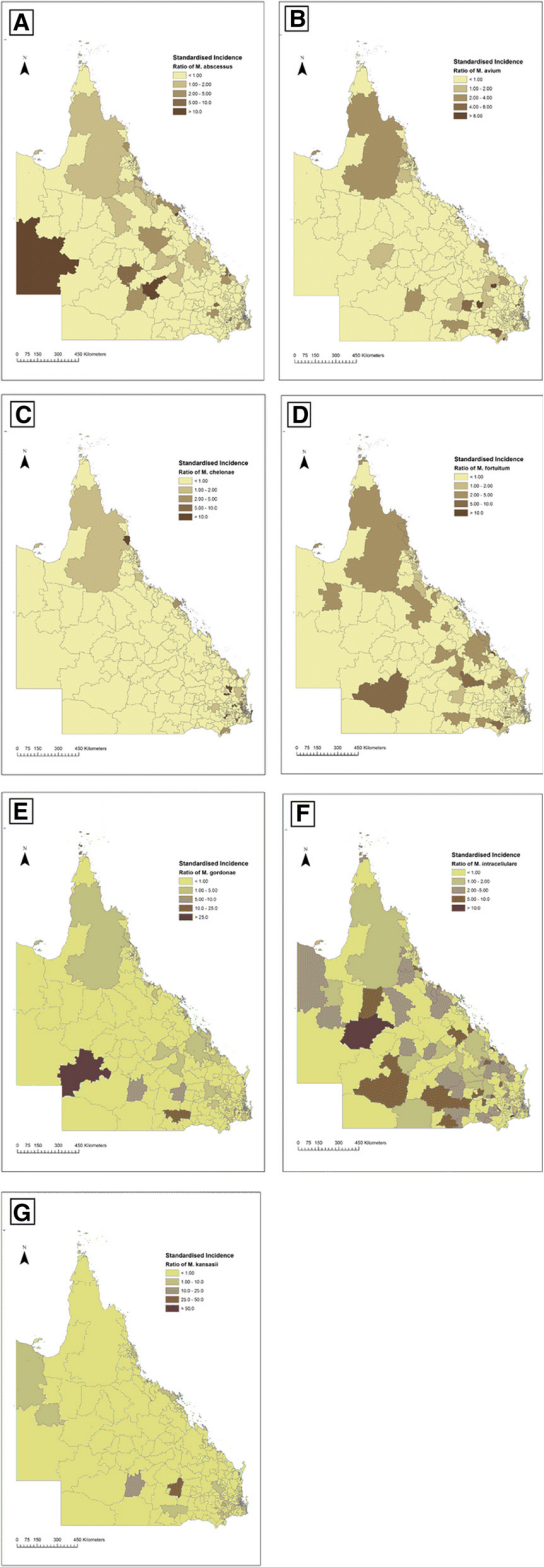
**The standardised incidence ratio for seven NTM species.** Standardised incidence ratios of *M. abscessus***(A)**, *M. avium***(B)**, *M. chelonae***(C)**, *M. fortuitum***(D)**, *M. gordonae***(E)**, *M. intracellulare***(F)** and *M. kansasii***(G)** from 2001-2011 mapped by postcode for Queensland, Australia.

The seven species that were most frequently isolated were selected for analysis. *M. intracellulare* accounted for nearly 35% of the NTM isolates. Isolates from extrapulmonary sites comprised 19.6% of isolates. Only in *M. fortuitum* and *M. chelonae* was the count higher in extrapulmonary than pulmonary sites. Maps of the SMRs for each NTM species by postcode are shown in Figure 1. Total counts were presented because previous studies have demonstrated no evidence that the strains causing pulmonary and extrapulmonary disease are different [16]. Each species of NTM showed distinct spatial patterns.

For four NTM species – *M. abscessus, M. avium, M. intracellulare* and *M. kansasii* – the models with the best fit (as determined using the DIC) included Models II and III – those with a spatially structured component, with Model III having the best fit for *M. intracellulare* and *M. kansasii* and Model II having the best for *M. abscessus* and *M. avium*. This indicated that risk of infection with these four NTM species demonstrated significant spatial heterogeneity after accounting for the covariates in the models. Furthermore, of these four species, significant covariates (soil depth, soil bulk density, earning < $52,000 and earning < $32,000) were found for *M. intracellulare* and *M. kansasii* (Table 2).

**Table 2:** **Modelling results for *M. intracellulare *** **and** ***M. kansasii***

**Model III**	***M. intracellulare***		***M. kansasii***	
**Variables**	**Posterior mean ± SD**	**RR (95% CI)**	**Posterior mean ± SD**	**RR (95% CI)**
**Intercept (α)**	-0.021 ± 0.014		-0.84 ± 0.37	
**Soil Depth (m)**	-0.13 ± 0.06	0.87 (0.78-0.97)		
**Earning < $52000**	0.19 ± 0.05	1.21 (1.09-1.34)		
**Soil Bulk Density (mg/m**^**3**^**)**			0.58 ± 0.18	1.79 (1.26-2.56)
**Earning < $32000**			-0.34 ± 0.16	0.71 (0.51-0.97)
**Heterogeneity *s *** **(structured)**	0.46 ± 0.12		1.57 ± 0.65	
**Heterogeneity *w *** **(unstructured)**	0.03 ± 0.03		0.13 ± 0.20	
**DIC**	6451.81		10123.70	

Among the other NTM species where the models did not support evidence of spatial heterogeneity (i.e. where the lowest DIC was associated with Model I), soil pH was identified as a significant predictor variable for *M. fortuitum* (RR 21%; 95% CI 1-45%) and soil nitrogen content was found to be a significant predictor variable for *M. chelonae* (RR 24%; 95% CI 3-50%).

Risk of *M. intracellulare* infection was estimated to increase by 21% per percentage increase of population earning under $52,000 and to decrease by 13% for every metre increase of topsoil depth. Risk of *M. kansasii* infection was estimated to increase by 79% per mg/m^3^ increase of soil bulk density and to decrease by 19% for every percentage increase in population earning under $32,000.

**Figure 2:**
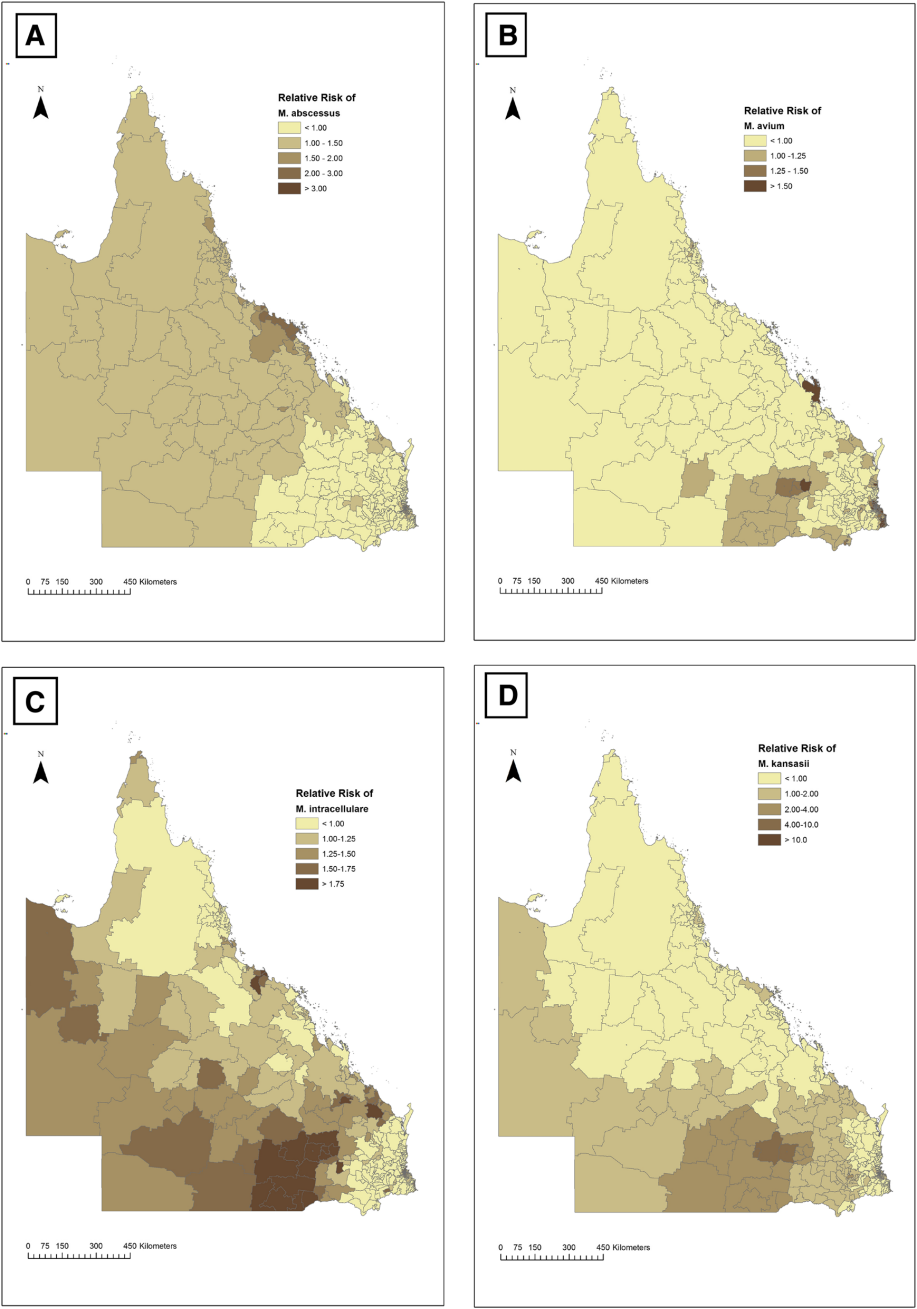
**The relative risk for four NTM species.** The relative risk for *M. abscessus***(A)**, *M. avium***(B)**, *M. intracellulare***(C)**, *M. kansasii***(D)** from 2001-2011 mapped by postcode for Queensland, Australia.

Figure 2 has four chloropleth maps that show the spatially-structured relative risk of *M. intracellulare*, *M. kansasii, M. abscessus and M. avium.* Each map shows the risk of each NTM species relative to no disease that may be attributed to spatial factors in the environment. Effectively, the maps identify ‘hotspots’ in the state. Clusters of high relative risk for *M. intracellulare* and *M. kansasii* were found in the Darling Downs region of south central Queensland. The cluster for *M. kansasii* was more geographically focused around the town of Roma. Notably, the relative risk was 75 times higher than the average (p < 0.05) for *M. kansasii* in the postcodes containing the towns of Clifford and Yuleba, nearby to Roma. There was also a cluster of high relative risk for *M. abscessus* in the Whitsunday region of Queensland.

The estimated variance of the spatial component was 0.46 ± 0.12, 1.57 ± 0.65 and 0.64 ± 0.18 for *M. intracellulare*, *M. kansasii* and *M. abscessus* respectively. This was comparatively larger than the variance of the unstructured components in these models, indicating that residual risk, after accounting for the covariates, was predominantly spatially structured.

## Discussion

Three clusters of postcodes with high risk for specific NTM infections were found in two distinct regions of Queensland. In addition, multivariate Bayesian analysis identified significant predictors of NTM risk, including socioeconomic and environmental factors, for four NTM species.

**Figure 3:**
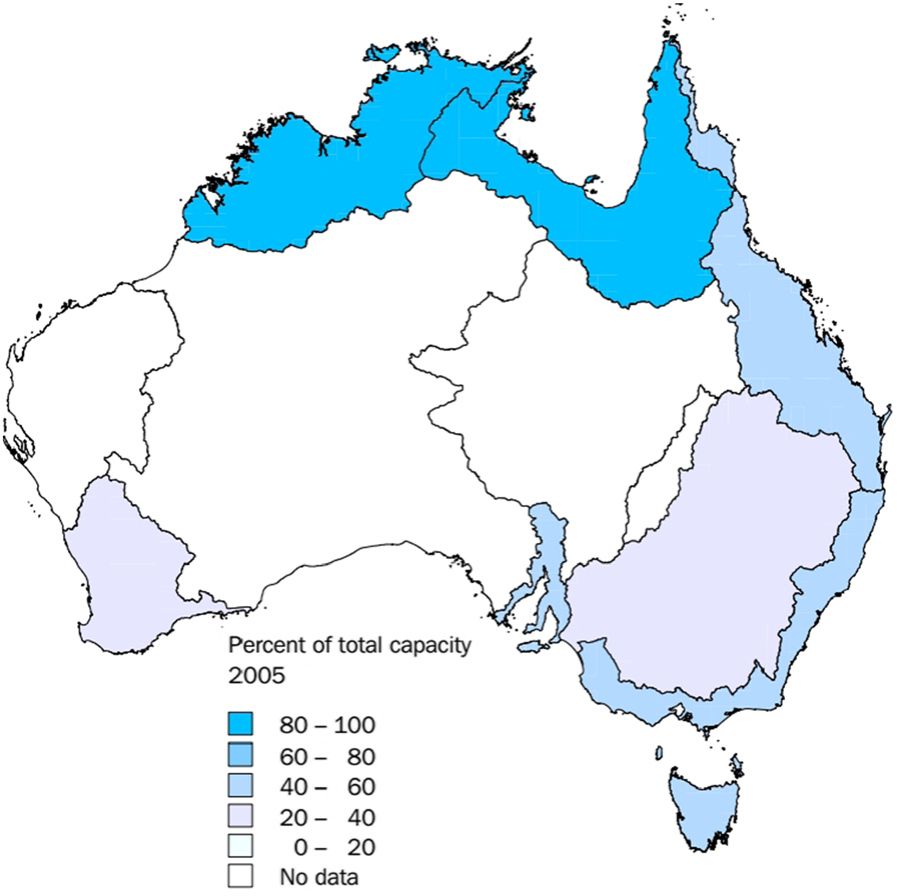
**Percent of total water capacity stored in large dams for each drainage division **[17]**.** Proportion of water stored in large dams for each Australian drainage divisions in 2005.

Geographically, the clusters of *M. intracellulare* and *M. kansasii* were located in a region overlying the Surat Division of the Great Artesian Basin. Interestingly, the western geologic border of the Surat Basin corresponds well with the western edge of the *M. intracellulare* cluster. This region is well known for its agricultural and mining activities with a number of developed petroleum and coal seam gas wells. In addition, the water supply for many communities in the region is primarily from private and communal bores, aquifers and rainwater tanks. It is difficult to ascertain the exact number and distribution of these water sources. However, Figure 3 shows the percentage of water in each region that is stored in large dams [17]. By inference, suburban households in areas surrounding these dams are more likely to source water from reticulated supplies rather than private bores.

There are several potential explanations for the significantly higher relative incidence of *M. kansasii* in the Yuleba and Clifford areas. Yuleba is a small town and the site of a major processing facility for silica deposits. Silicosis has been identified as a host susceptibility factor for *M. kansasii*. Moreover, mining activity is common in the Maranoa-Balonne region (the broader administrative area that includes Yuleba and Clifford). There have been several studies linking *M. kansasii* to industrial activities such as gold mining and iron manufacture in South Africa, Japan and the former Czechoslovakia [18-20]. In the study by Corbett and Hay, new pulmonary cavitations from *M. kansasii* were more likely to be found in miners with previous TB scarring or silicosis. In addition, Yuleba has had a reticulated supply from bore water since the 1960s. *M. kansasii* has been identified as a cause of opportunistic infections from drinking water distribution systems [21-23]. Nonetheless, while *M. kansasii* has been found in potable water supplies in the greater Brisbane metropolitan area [2], relatively few strains match clinical isolates from patients [24,25].

The evidence linking reticulated water as a source of infection with *M. intracellulare* is scant [25,26]. As sequencing technologies have become more refined, *M. intracellulare* previously identified in water and biofilm samples have actually been found to be *M. chimaera*. In fact, older studies from QLD have found *M. intracellulare* in soil, house dust and rainwater tanks [27,28]. Synthesizing these perspectives, transmission of *M. intracellulare* may be more related to dust inhalation or direct ground water contamination by soil. Contamination during run-off events is also possible and this has been previously postulated as a potential mechanism for another species of NTM [29]. The co-incidence of high relative risk in Yuleba for both *M. kansasii* and *M. intracellulare* may indicate common routes of exposure potentially mediated through inhalation of dust or fine soil particles or contamination of water by these two species.

The last report relating *M. kansasii* to soil appears to have been published over 40 years ago [30]. More recently, an association was reported between *M. intracellulare* and anaerobic soils [31,32]. Another possible route of NTM transmission in the Maranoa region, where there is greater reliance on tank and bore water, is soil contamination of water sources. Here we found that risk of *M. intracellulare* infection was correlated with a shallower soil depth. Potential reasons may include the poor rooting of vegetation in shallow soil, leading to decreased uptake of soil nutrients, leaving a nutrient rich topsoil environment in which mycobacteria may thrive [33]. Another reason may be human activities related to shallow soil in agricultural regions such as the Darling Downs. A shallow soil depth is usually associated with low Plant Available Water Capacity (PAWC) and low crop yields [34]. To improve yields, soil is often disturbed in a process called “deep ripping”. This activity may aerosolise soil particles. Conversely, another potential response to improve crop yields in shallow soils is liming - this may actually inhibit mycobacteria growth as it increases acidity. Liming can be done before, during or after deep ripping.

Soil bulk density, found to be associated with risk of *M. kansasii* infection, is the dry weight of soil per m^3^ of soil volume. This variable can be affected by a number of other soil factors such as permeability, composition and depth. Like soil depth, soil bulk density is affected by human activity. Notably, soil bulk density can be increased by constant or inappropriate tilling as well as by compressive forces from heavy agricultural machinery [35]. These activities may also increase the risk of mycobacteria exposure from soil. Increased bulk density impairs root growth, further contributing to nutrient rich topsoil environments that are conducive to mycobacterial growth [33]. Peat soil composition was found to be a predictor of NTM risk in other studies but this is unlikely to be the case in Australia, because peat soil is only found in sparse Alpine regions of Australia [36,37].

The variable for proportion of population earning < $32,000 may be considered a representative indicator of socioeconomic status of the postcode. The finding of an increased relative risk for *M. kansasii* infection in postcodes with a higher socioeconomic status is consistent with the literature comparing NTM and socioeconomic determinants [38]. The positive correlation between *M. intracellulare* and proportion of the population earning *<* $52,000 is harder to interpret. There are a number of potential confounders of this association, including smoking, alcohol intake and likelihood of seeking medical attention, that need to be considered.

The cluster of *M. abscessus* in the Whitsunday region requires further investigation. The region is well known for its tropical climate and pristine marine environments, and is a popular tourist destination. Isolates of *M. abscessus* from swimming pools and rainwater tanks have been linked to patient isolates in Brisbane [39]. Jacobs et al [40] associated water characteristics in a coastal lagoon with NTM. They found positive correlations with water temperature, nitrogen and phosphorus content and negative correlations with depth and salinity. Soil nitrogen and phosporus content was not associated with *M. abscessus* in our analysis.

While individual mycobacterial species have their own preferred growth environments [41], the lack of consistency in significant predictor variables across the seven NTM species analysed highlights the differing epidemiology of these organisms. Using aggregated NTM data by postcode over a 10 year period may have masked associations between NTM and environmental, climate and socioeconomic variables at finer spatial and temporal resolutions. The artificial nature of postcode boundaries may also have affected observed associations and spatial patterns, and might have given rise to ecological fallacy, whereby associations observed at the scale of the postcode do not reflect the association between exposure of individuals to a risk factor and their resultant risk of infection [42,43].

Furthermore, identification of important predictors is also likely to be made more challenging by the multi-year latency periods of slow growing NTM species. Changing climactic conditions in Queensland add an additional layer of complexity: the 10-year period of the study has been Australia’s hottest decade on record [44]. In Queensland, it was marked by severe drought conditions culminating in statewide water restrictions. This was followed by the wettest year on record in 2010, with significant flooding occurring twice that year. The first flood occurred in the central QLD regions where clusters of NTM have been identified. The second flood affected the state more widely. This complexity makes any analysis of secular trends during this period difficult to interpret. Finally, issues such as the presence of petroleum wells identified in our analyses require further investigation.

## Conclusions

Distinct spatial clusters of *M. kansasii, M. intracellulare* and *M. abscessus* were identified in regions that contribute to Australia’s agricultural, mining and tourism industries. Additionally, a number of socio-ecological, economic and environmental factors were found to be associated with NTM infection risk. A better understanding of the epidemiology of NTM infections could form the basis of cost-effective and targeted public health initiatives for the control of this disease in Australia and globally.

## Competing interests

All of the authors declare no financial, professional, or otherwise personal interest of any nature or kind in any related product, service, and/or company.

## Authors’ contributions

ACAC conceived and designed the study. RMT was involved in the clinical data collection. MPC prepared datasets and statistical analyses. MPC drafted the manuscript and all authors contributed substantially to the revision of the manuscript. All authors read and approved the final manuscript.

## Pre-publication history

The pre-publication history for this paper can be accessed here:

http://www.biomedcentral.com/1471-2334/14/279/prepub
